# Differential Response in Downstream Processing of CHO Cells Grown Under Mild Hypothermic Conditions

**DOI:** 10.1002/btpr.1726

**Published:** 2013-05-02

**Authors:** Andrew S Tait, Richard D R Tarrant, M Lourdes Velez-Suberbie, Daniel I R Spencer, Daniel G Bracewell

**Affiliations:** Dept. of Biochemical Engineering, Advanced Centre for Biochemical Engineering, University College LondonTorrington Place, London, WC1E 7JE, U.K.; Ludger Ltd, Culham Science CentreAbingdon, Oxfordshire, OX14 3EB, U.K.

**Keywords:** mild hypothermic culture, apoptosis, cell cycle, downstream processing, host cell proteins

## Abstract

The manufacture of complex therapeutic proteins using mammalian cells is well established, with several strategies developed to improve productivity. The application of sustained mild hypothermic conditions during culture has been associated with increases in product titer and improved product quality. However, despite associated cell physiological effects, very few studies have investigated the impact on downstream processing (DSP). Characterization of cells grown under mild hypothermic conditions demonstrated that the stationary phase was prolonged by delaying the onset of apoptosis. This enabled cells to maintain viability for extended periods and increase volumetric productivity from 0.74 to 1.02 g L^−1^. However, host cell proteins, measured by ELISA, increased by ∼50%, attributed to the extended time course and higher peak and harvest cell densities. The individual components making up this impurity, as determined by SELDI-TOF MS and 2D-PAGE, were shown to be largely comparable. Under mild hypothermic conditions, cells were less shear sensitive than those maintained at 37°C, enhancing the preliminary primary recovery step. Adaptive changes in membrane fluidity were further investigated by adopting a pronounced temperature shift immediately prior to primary recovery and the improvement observed suggests that such a strategy may be implementable when shear sensitivity is of concern. Early and late apoptotic cells were particularly susceptible to shear, at either temperature, even under the lowest shear rate investigated. These findings demonstrate the importance of considering the impact of cell culture strategies and cell physiology on DSP, by implementing a range of experimental methods for process characterization. © 2013 American Institute of Chemical Engineers *Biotechnol. Prog*., 29:688–696, 2013

## Introduction

The demand for therapeutic proteins produced by mammalian cell lines, such as Chinese hamster ovary (CHO) cells, continues to grow. Significant achievements in process optimization have been made to meet the clinical demands of these proteins, which can have very high dosing regimes, often of more than 1 g per dose.^1^ These include incorporation of enhanced cell line selection technologies, development of media and feeds, and scale-up to bioreactor volumes of up to 25,000 L.^2^ Despite these improvements additional strategies have been adopted to increase volumetric productivities, extending the use of existing manufacturing facilities and reducing the capital expenditure associated with building and validating new facilities or outsourcing to contract manufacturer organizations. These have included the optimization of dissolved oxygen tension (DOT), pH, and temperature, all of which have been shown to have an influence cell metabolism, productivity, and product quality.^3^–^5^ The use of mild hypothermic conditions, by lowering the culture temperature to below the normal physiological temperature of 37°C, to enhance cell specific productivity of recombinant proteins, has been extensively studied.^5^–^8^ It should also be considered that dissolved oxygen concentration increases as temperature is reduced.^3^,^9^ However, the effect of decreasing the culture temperature on downstream processing (DSP) performance and host cell protein (HCP) profile has yet to be considered.

Lowering culture temperature is a strategy used to control cell proliferation during cell culture and is typically employed during the mid or late exponential growth phases.^10^ Reducing the culture temperature arrests cells in the G1-phase of the cell cycle when the cells are larger^11^ and have a lower glucose consumption and oxygen uptake and lactate and ammonia production than nonarrested cells.^9^,^10^ Mild hypothermic conditions also have the advantage of extending the stationary phase and maintaining higher cell viability,^8^ improved product quality^3^,^12^ and decreasing the sensitivity to apoptotic agents.^13^

It has been reported that the effect of lowering culture temperature on cell specific productivity is cell line dependent.^14^ In some cases increases in cell specific productivity^5^–^7^ have been observed whilst in others the productivity decreases or remains comparable to nonarrested cells.^9^,^14^

The glycosylation profile of recombinant proteins can be affected by the culture conditions, such as pH, temperature, culture media and mode of operation.^15^,^16^ It has been suggested that reducing culture temperature helps to retain the glycoform, as high cell viability is maintained throughout culture and the release of glycosidases, which target monosaccharides from the glycan, is minimized.^7^,^15^

Although a number of studies have been undertaken on mild hypothermic conditions, these have largely focussed on understanding the physiological response to this stress and on the identification of key biomarkers associated with it. In line with the quality by design (QbD) measures being adopted industry-wide further understanding of the effects of implementing strategies such as mild hypothermic conditions at process scale, on both the manufacturing process and the target product itself is becoming increasingly important to regulators.^17^ To assist with such activities, the availability of scale-down methodologies which accurately mimic process scale operations for process development and validation purposes has become paramount. Until recently much of this work has focussed on cell culture, including high throughput screens in microwell plates and miniature bioreactor formats^18^ or on purification process steps in geometrically scalable chromatography columns or other alternative scale-down formats.^1^ As these tools continue to evolve, integration of primary recovery is being seen as increasingly important in the development process, to understand its impact on the release of process related impurities. This is being achieved by the development of a number of scale-down methodologies for these process operations, including for centrifugation,^19^,^20^ microfiltration,^21^ and depth filtration.^22^

In this study, we examine the effect of mild hypothermic conditions on an industrially relevant IgG_4_ producing GS-CHO cell line grown in monitored and controlled stirred tank bioreactors (STRs), operated under fed-batch mode with serum free media. Using flow cytometry-based assays, we investigate the physiological effects of mild hypothermic conditions on the cells at certain time-points during fermentation. We have previously demonstrated that cell physiology can play an important role in primary recovery performance, particularly during operations such as centrifugation, which is associated with the high levels of energy dissipation that can cause cellular damage during processing.^23^,^24^ We have therefore considered the potential impact of these cellular responses on the DSP. First, we evaluate differences in the impurity profile challenging the purification process by using a variety of analytical techniques. We then examine the impact on centrifugation performance by using an ultra scale-down (USD) methodology.

## Material and Methods

### Cell culture

Experiments were carried out using a Glutamine synthetase Chinese hamster ovary (GS-CHO) cell line stably expressing an IgG_4_ monoclonal antibody (CY01 cell line, generously provided by Lonza Biologics, Slough, UK), as described in Velez-Suberbie et al.^25^ Two bioreactors were run in parallel with working volumes of 3.5 L and set points of pH 7.1 and DOT 30% of air and were inoculated simultaneously using the same seed culture. Both bioreactors were operated at 37°C until late exponential phase (day 6) when the temperature was shifted to 32°C in one bioreactor and maintained for the duration of the culture.

For investigating reduced processing temperature, samples were taken from a fed-batch bioreactor run, which was performed as described above, under standard conditions. Samples were taken at high and low viabilities and either immediately used at culture temperature for USD shear and centrifugation or adjusted to 4°C and held for 5 h, prior to processing.

### USD shear and centrifugation studies

A rotating shear device, mimicking the shear typically experienced in the feed zones of industrial centrifuges and a 96-well plate bench-top centrifugation method, providing a range of clarification conditions representing a range of volumetric flow rates at process-scale were used as described previously.^23^

## Analytical Techniques

### N-glycan release, labeling, clean-up, and analysis

Supernatant from samples was taken on days 10 and 12 (37°C) and 14 and 20 (32°C) and purified and prepared for mass spectrometry analysis as described previously in Velez-Suberbie et al.^25^

### Apoptosis staining

Progression into apoptosis was determined from day 8 onwards by staining with a commercially available annexin V-FITC/7ADD kit (Invitrogen, Paisley, UK) and analysis using a Coulter Epics XL-MCL Flow Cytometer (Beckman Coulter, High Wycombe, UK). Cell culture samples were prepared following Manufacture's protocol, analyzed using 488 nm excitation and detected using 525 and 675 nm band-pass filters for annexin V and 7-ADD, respectively, and collected for 300 s or 10,000 events. All samples were analyzed within 30 min of staining. Positive control samples were analyzed to ensure appropriate gating was applied.

### Cell cycle analysis

Cell cycle distribution was determined from day 8 onwards using propidium iodide staining combined with flow cytometry (Coulter Epics XL-MCL, Beckman Coulter). Cell culture samples were centrifuged (Eppendorf 5415, Cambridge, UK) at 500*g* for 5 min at 20°C, the supernatant discarded and the cell pellet washed in PBS. The suspension was centrifuged again at 500*g* for 5 min at 20°C, the supernatant was discarded and the cell pellet resuspended in 70% ice cold ethanol to a concentration of 5 × 10^6^ cells mL^−1^. Cells were fixed for 30 min at 4°C, and then centrifuged at 1500*g* for 5 min at 20°C. The supernatant was discarded and the pellet was resuspended in 0.5 mL of PBS. The cell suspension was centrifuged at 800*g* for 5 min at 20°C, the supernatant discarded, 75 µL of a 100 µL mL^−1^ of Ribonuclease A solution (Sigma-Aldrich, Gillingham, UK) was added and incubated for 5 min at room temperature. Propidium iodide (0.75 mL of a 50 µL mL^−1^ solution) was added and incubated for 5 min at room temperature (Sigma-Aldrich). The samples were analyzed by flow cytometry (Coulter Epics XL-MCL, Beckman Coulter) using 488 nm excitation and a 675 nm band-pass filter for detection. To obtain the cell cycle distribution, the histogram data file generated was analyzed using the Cylchred program (Cardiff University, Cardiff, UK).

### Particle size distribution analysis

A CASY analyzer (Innovatis, Bielefeld, Germany) was used to determine the particle size. The CASY was used with a 150 µm orifice and set to measure up to 40 µm with a 5 times repeat measurement, from which an average was reported. The particle volume was calculated from the diameter determined, assuming all particles were spherical. The volume was then determined as a percentage of the entire volume of material present.

### Product concentration by HPLC analysis

The mAb concentration was determined by protein G-HPLC analysis using an Agilent 1200 HPLC (Agilent Technologies, South Queensferry, UK). Samples (100 µL) were loaded onto a 1 mL HiTrap protein G column (GE Healthcare, Pittsburgh, PA), washed with 20 mM sodium phosphate, pH 7.0 and eluted using 20 mM glycine hydrochloride, pH 2.8, with absorbance measured at 280 nm. The product peak was integrated and the concentration determined using a standard curve of purified mAb of known concentration.

### HCP ELISA

HCP concentration was determined using a commercially available microtiter sandwich ELISA (Cygnus Technologies, NC), as described previously.^26^

### Surface-enhanced laser desorption ionization: time of flight (SELDI-TOF) mass spectrometry

Samples were prepared and analyzed as previously described,^26^ using normal phase SELDI chips (NP20, Bio-Rad Laboratories, Hemel Hempstead, UK).

### 2D-Polyacrylamide Gel Electrophoresis (2D-PAGE)

Samples from the relevant timepoints of each culture were prepared and Isoelectric focussing and SDS-PAGE performed as previously described.^26^ The gels were then stained with Sypro Ruby (Invitrogen), according to the manufacturer's protocol and scanned using a Typhoon 9400 laser scanner (GE Healthcare), with the following settings; 600 V PMT, 532 nm (green laser) for excitation and 610BP30 emission filter and a pixel size of 100 µm.

Progenesis Samespots software was used for spot comparison (version 4.0, Nonlinear Dynamics, Newcastle upon Tyne, UK). After selecting an appropriate reference image the gels were aligned by adding manual vectors and then further aligned with additional automatic vectors. All images were selected for further processing and all detected spots subsequently normalized. Gel images were then divided into the two experimental groups and compared using the between-subject-design analysis function. Detected spots were reviewed on a one to one basis and adjusted where appropriate (e.g., splitting and merging of spots). Comparison between the two groups was made, using the software to perform an ANNOVA test and generate *P* values, power values and fold change differences of selected spots. Only spots with *P* values of <0.05 and power values of >0.8 were selected. Any spots identified in areas of the gels masked by mAb heavy or light chains in any of the samples analyzed were discounted.

## Results and Discussion

In the following sections, we discuss the implications of implementing mild hypothermic conditions, a commonly used cell culture strategy to improve product titer, on the subsequent stages within DSP. We first examine the change in physiology that occurs to CHO cells grown under mild hypothermic conditions (32°C) and then go on to investigate how the change in culture conditions can also affect the impurity profile of the process stream, specifically the HCP population. Finally, we evaluate how the physiological changes associated with mild hypothermic conditions can affect the shear susceptibility of cells and as a consequence the performance of process-scale centrifugation, which is commonly used for primary recovery in large-scale biopharmaceutical manufacture.

### The physiological response of GS-CHO cells to mild hypothermic conditions

**Figure 1:**
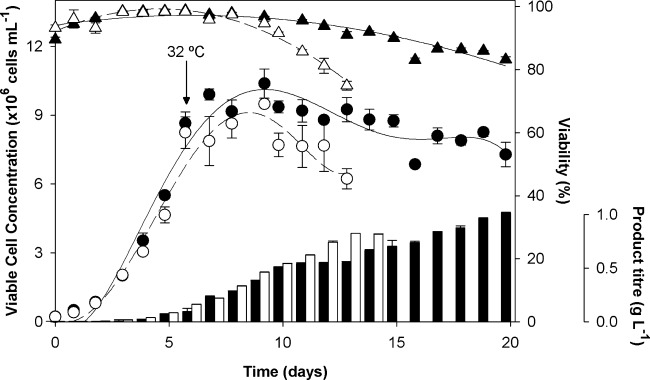
Growth profiles of mAb producing CHO cells (GS-CY01) grown under standard and mild hypothermic conditions (32°C) Cultures were grown in parallel STRs (3.5 L working volume) with a temperature change from 37 to 32°C implemented in one bioreactor on day 6 of culture. The viable cell concentration (• 32°C; ○ 37°C) and viability (▲ 32°C; Δ 37°C) were determined. *n* = 3 (replicate cell counts) ± s.d. Product titer was determined by protein G HPLC (▪ 32°C; □ 37°C).

**Figure 2:**
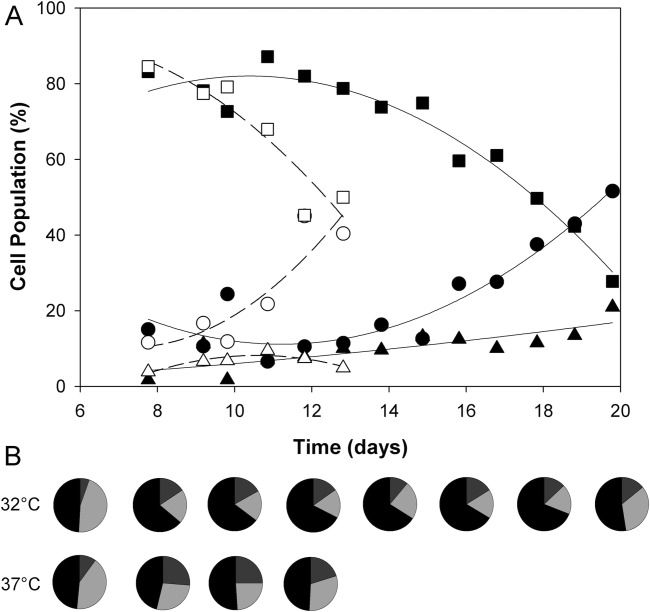
Effect of mild hypothermic (32°C) culture conditions on cell physiology Cultures were grown in parallel STRs (3.5 L working volume) with a temperature change from 37 to 32°C implemented in one bioreactor on day 6 of culture. A: Apoptosis was determined by flow cytometry for samples taken from the cultures at 32°C [viable (▪,—), early apoptotic (▲,—), and late apoptotic cells (•,—)] and 37°C [viable (□,- -), early apoptotic (Δ,- -), and late apoptotic cells (○,- -)]. The trend lines represent second order linear regressions. B: The cell cycle distribution was determined by flow cytometry and the percentage in G1 (▪), S (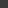), and G2/M (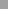) subpopulations was calculated.

**Figure 3:**
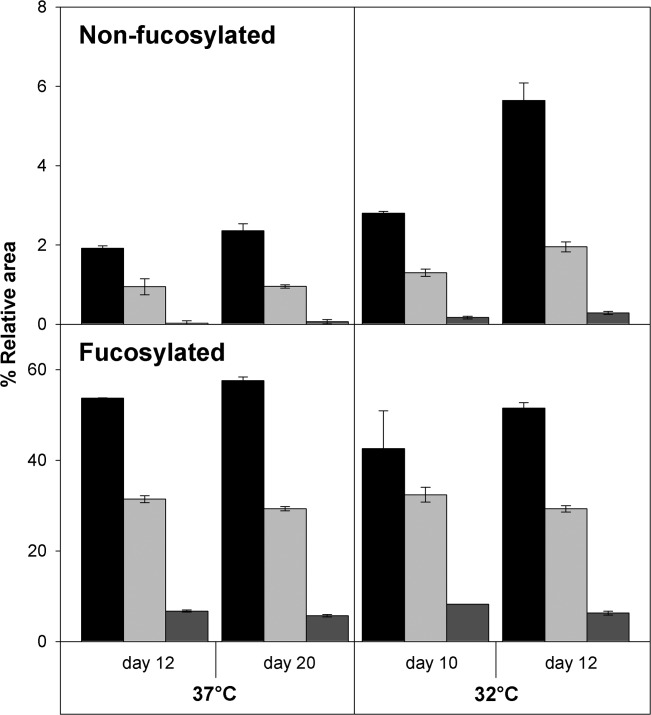
Glycosylation profiles of mAb producing CHO cells (GS-CY01) grown under standard and mild hypothermic conditions (32°C) Cultures were grown in parallel STRs (3.5 L working volume) with a temperature change from 37 to 32°C implemented in one bioreactor on day 6 of culture. Samples removed on specified time points were purified by protein A chromatography and the glycosylation profiles were determined, % relative area of fucosylated and non-fucosylated glycans (▪ G0(F); 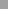 G1(F); 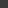 G2(F). *n* = 3 (replicates of antibody sample) ± s.d.

To investigate the effect of mild hypothermic conditions (32°C) on mammalian cells grown in suspension, two 5 L fed-batch cultures were grown in parallel using a model mAb producing GS-CHO cell line. Operating conditions were matched in each bioreactor until a cell density of 8 × 10^6^ cells mL^−1^ was obtained (day 6), indicating an end to the exponential phase, at which point the temperature of one reactor was shifted from 37°C to 32°C until harvest. As has been demonstrated previously,^5^–^8^,^27^ the application of mild hypothermic conditions prolonged the stationary phase compared with the culture maintained at 37°C (Figure 1), and cell viability was maintained above 80% until day 20 of culture, whereas viability dropped to below 80% on day 12 under standard culture conditions. Further analysis of the cells demonstrated that this prolonged stationary phase delayed apoptosis progression (Figure 2A) and increased the proportion of cells within the G1 phase of the cell cycle from 46 to 64%, which is consistent with previous studies (Figure 2B).^5^–^8^,^10^,^12^ The application of mild hypothermic conditions can increase final product titer and is observed to do so here with an increase at harvest point from 0.74 g L^−1^ at 37°C to 1.02 g L^−1^ at 32°C. An increase in cell specific productivity, which is also often associated with mild hypothermic culture conditions,^14^ was not observed (Table 1). However, it did allow the culture duration to be extended significantly and increase the product titer on day of harvest. These findings were in agreement with previous work performed in shake flasks, operated in batch mode (data not shown). Increased cell specific productivity is known to be a cell line specific phenomenon; however, it is interesting to note that cells cultured at 32°C showed a gradual increase in cell size during culture and that when this is taken into account, the volume specific productivity at time of harvest approaches that observed in cells grown at 37°C. The effect of mild hypothermic conditions on product quality was determined on days 12 and 20 for 32°C and days 10 and 12 for 37°C (Figure 3) by N-glycan analysis. It showed that decreasing the culture temperature did not impact on the glycosylation profile, whilst the length of culture had an impact of the ratio of the glycosylated species, in agreement with findings by Reid et al.2010 As the culture progressed, there was an increase in relative abundance of short glycans (G0F) 48 to 52% and 53 to 58% for 32 and 37°C, respectively, and a decrease on the proportion of long glycans (G1F/G2F) 41 to 36% for 32°C and 38 to 35% for 37°C.

**Table 1:** Specific Productivity, Volume Specific Productivity, and Cell Size from CHO Cells (GS-CY01) Grown Under Mild Hypothermic and Standard Conditions

Time (days)	Average Cell Specific Productivity (pg cell^−1^ d^−1^)		Average Volume Specific Productivity (pg mm^−3^ d^−1^)		Average Cell Size (µm)	
	32°C	37°C	32°C	37°C	32°C	37°C
0–6	7.1	12.6	1.23 × 10^4^	2.10 × 10^4^	15.0	14.9
7–13	6.5	12.7	1.57 × 10^4^	3.03 × 10^4^	16.1	16.5
14–20	7.7	N/A	2.68 × 10^4^	N/A	18.3	N/A
Overall	7.2	11.8	1.92 × 10^4^	2.78 × 10^4^	16.5	15.7

N/A: Bioreactor was harvested on day 13.

### Analysis of the HCP distribution

The HCP profile has been shown to be affected by physiological differences prior to and during primary recovery^24^,^29^ and differential expression of specific proteins in response to this state may mean that the make-up of the impurity profile is changed. For these reasons, the effect of mild hypothermic conditions on the HCP content was investigated. The HCP content determined by an industry standard ELISA assay^30^ was ∼50% higher in the culture grown under mild hypothermic conditions, raising from 101,741 ± 13,211 ppm (ng per mg antibody) at 37°C to 148,264 ± 13,610 ppm at 32°C, measured on days 12 and 20, respectively (Table 2). Under standard conditions the majority of HCPs from the cell line used in this work have been shown to be intracellularly located, arising from lysed or fragmented cells during culture.^24^ The higher peak and harvest viable cell counts and extended culture period associated with the culture grown under mild hypothermic conditions are therefore likely to be significant contributing factors accounting for this difference.

**Figure 4:**
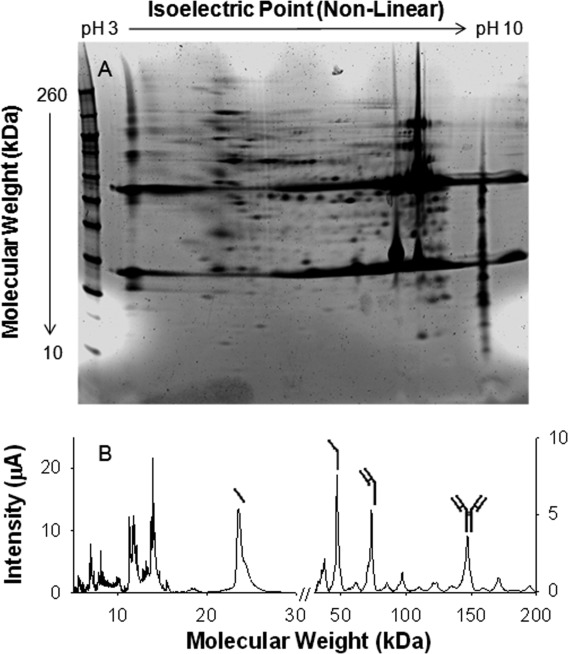
Representative example of a 2D-PAGE gel image (A) and SELDI-TOF MS spectra (B), from the fed-batch culture operated at 37°C For 2D-PAGE, pH 3–10 nonlinear IPG strips and 4–12% Bis-Tris SDS-PAGE gel were used, with a total protein load of 200 µg. All samples were run in triplicate and stained with Sypro Ruby. For SELDI-TOF MS, analysis was performed using normal phase chips, with all samples run in triplicate. Peaks attributed to be product related species are indicated.

The differences in proportion of HCPs may be also due to differential expression of these impurities as a result of implementing hypothermic conditions during culture.^31^ As this strategy has been adopted here and in light of differences in abundance as determined by HCP ELISA two complementary proteomic techniques, SELDI-TOF MS and 2D-PAGE, were used to examine the individual components of this impurity population (Figure 4). SELDI-TOF-MS has been used previously to examine HCP expression throughout culture^24^,^32^ and HCPs during purification,^26^ but only now to examine differences as a result of physiological stress. 2D-PAGE is an extensively used proteomic technique and can be coupled with mass spectrometry for identification and has been used in a number of studies to focussing on HCPs.^22^,^24^,^29^

**Table 2:** HCP Analysis Under Standard and Mild Hypothermic Conditions (32°C)

	32°C	37°C
HCP ELISA (µg HCP mg^−1^ mAb)	148 ± 14	102 ± 13

HCP quantification was conducted using a commercially available sandwich ELISA kit. The data shown is the average of triplicate replicates within the range of the assay, normalized to the mAb concentration.

For SELDI-TOF MS, chips with normal phase chemistry were used to bind the majority of proteins present. An example of the typical mass spectra output generated is shown in Figure 4. Many of the prominent peaks are attributed to be product related, or associated fragmented and multiply charged species, however, in the lower mass range (<20 kDa) many of the peaks are likely to be HCPs. Comparison of the individual peaks observed within this range showed that the majority of peaks had comparable areas, but there were a small proportion of peaks having statistically significant differences. Analysis by 2D-PAGE (Figure 4) provided further evidence of minor differences in some individual HCPs, although again the majority of spots had comparable volumes.

Although the proteomic methods used in this work suffer from some limitations, as discussed in further detail in Tarrant et al.,^26^ any differences in the impurity profile, even if minor, may be significant as these are one of the primary impurities targeted for removal during the purification process.^33^ This also provides rationale for further examination of HCPs, when changes to the existing production process are made, in particular on whether or not there is a consequence on the effectiveness of the purification process. Improvements in sample preparation (e.g., use of immunodepletion), implementation of alternative proteomic techniques such as 2D LC-MS or examination of these impurities at the individual component level (e.g., for HCPs which are shown to be product associating, have protease activity or are associated with particular safety concerns) would therefore greatly benefit efforts in future process development and improve current process understanding.

### Implications of mild hypothermic conditions on DSP

The benefits of high recombinant protein titer and product quality associated with mild hypothermic conditions are well established.^9^ It is also known (and demonstrated here) that the implementation of such strategies and other changes in bioreactor conditions can have a dramatic effect on the physiological state of the cells^34^,^35^ and that such changes can in turn alter the way in which cells respond to shear forces. In addition, the shear experienced in DSP has also been well characterized and shown to yield energy dissipation rates many times higher than those typically seen in bioreactors,^36^ which can have significant effects on the cell containing culture medium.^23^,^24^,^37^ Here, we investigate the impact upstream process conditions can have on the response of cells to the shear forces experienced in DSP; specifically, disc-stack centrifugation, which is typically the first recovery stage in large-scale mammalian cell culture. To enable a more comprehensive study of different centrifugation conditions, we employed an USD mimic of process scale centrifugation, which has been previously used to evaluate this cell line.^23^,^24^

Cell culture material taken from bioreactors either controlled at 32 or 37°C were exposed to a range of shear conditions that mimic those that would typically be experienced during primary recovery using large-scale centrifugation. To compare the performance from either culture the viability of the cells pre-shear and post-shear was determined and the clarification performance measured using an USD centrifugation mimic.^23^ Shear sensitivity of CHO cells has previously been demonstrated to be associated with the viability of the culture.^23^,^24^ However, the implementation of mild hypothermic conditions prolongs the period over which cells maintain a high viability; therefore to ensure the response to DSP observed is a fair comparison, cultures with the same viability rather than those taken on the same day were compared, that is, days 10 (91.6%) and 12 (81.2%) for 37°C and 14 (91.9%) and 20 (83.2 %) for 32°C cultures.

**Figure 5:**
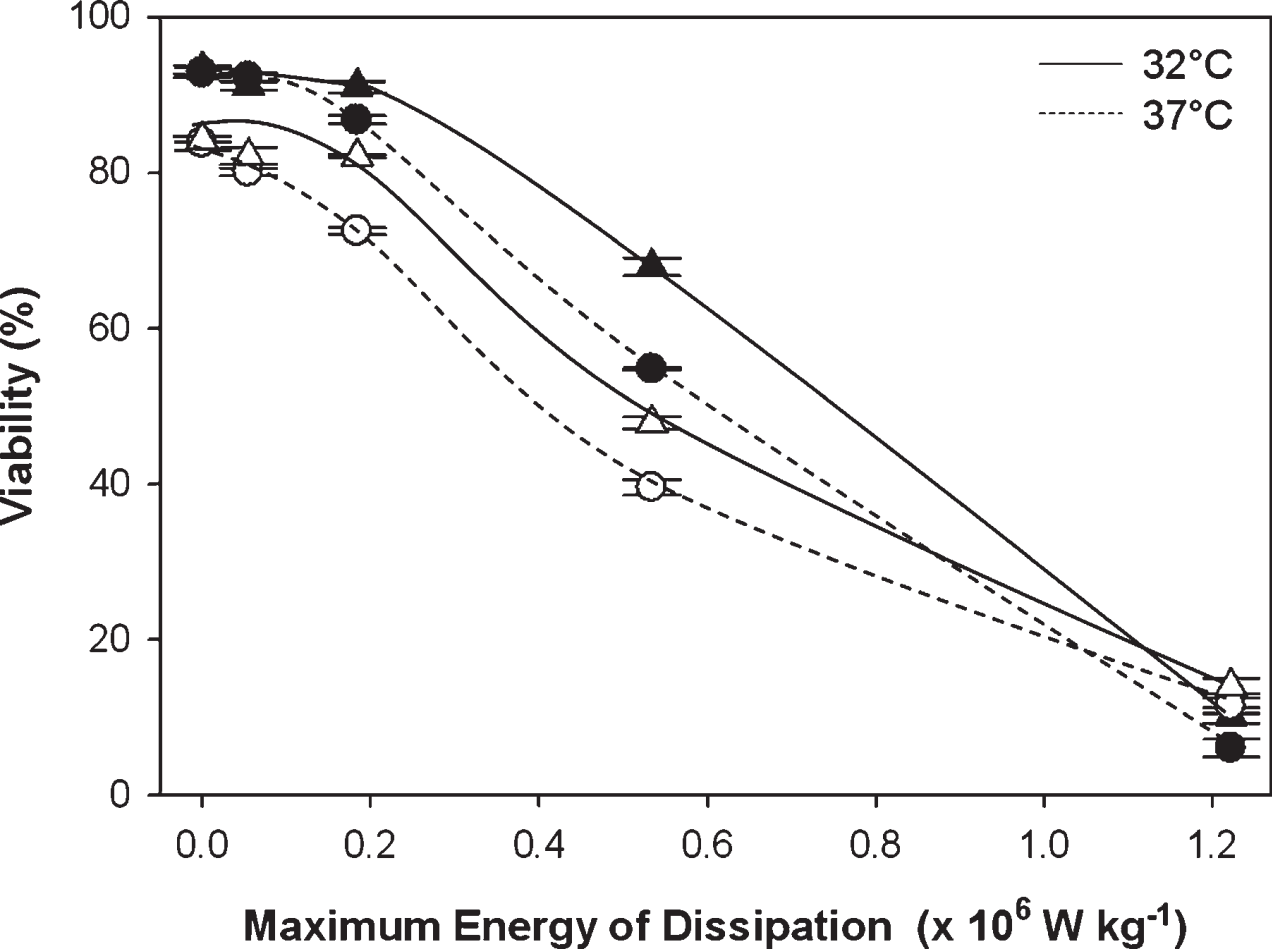
Effect of mild hypothermic growth conditions on the response of CHO cells to shear Samples taken from fed-batch cultures operated at 32 or 37°C were exposed to shear environments that represent those typically experienced in industrial scale centrifuges. The viability determined prior to and after application of the shear using a Vi-Cell XR. The plots depicted here show the results from samples that had the same pre#x02010;shear viability, represented here as high [37°C, day 10 (•); 32°C, day 12 (▲)] and low [37°C, day 12 (○); 32°C, day 20 (Δ)]. *n* = 3 (repeat viability measurements) ± s.d.

The reduction in culture viability with the implementation of increasing energy can be considered a measure of the shear sensitivity of the culture. When cultures taken from mild hypothermic conditions and standard growth conditions are compared (Figure 3), both cultures demonstrate an energy dissipation threshold (0.195 × 10^6^ W kg^−1^) above which a significant reduction in viability is observed. However, cells grown under mild hypothermic conditions show a smaller reduction in viability indicating these cells are more shear resistant. It has been suggested that the shear sensitivity of cells relates to the stage of the cell cycle they are in, with those in the G2 phase being larger and therefore more sensitive.^35^ However, we observed that when the viability of the culture is taken into account, it is the culture conditions and not the cell size being the predominant factor in shear sensitivity. Cells that were harvested at a high viability were observed to be the same average diameter (16.46 µm at 32°C; 16.39 µm at 37°C) but showed a different reduction in viability (Figure 5). Those harvested at a low viability where the cells grown at 32°C were observed to be larger (19.71 µm at 32°C; 17.52 µm at 37°C) also showed a greater resistance to shear. Addition of cell culture components such as Pluronic F-68 have been shown to increase membrane rigidity and as a result reduce shear sensitivity.^38^ The cell membrane can also be modified due to homeoviscous adaption to mild hypothermia, in which the activation of specific cell signaling pathways enables changes in the lipid composition to take place.^39^,^40^ The data presented therefore indicates that the implementation of mild hypothermic conditions results in cells that are more resistant to the hydrodynamic conditions experienced in DSP, regardless of the difference in cell size observed.

**Figure 6:**
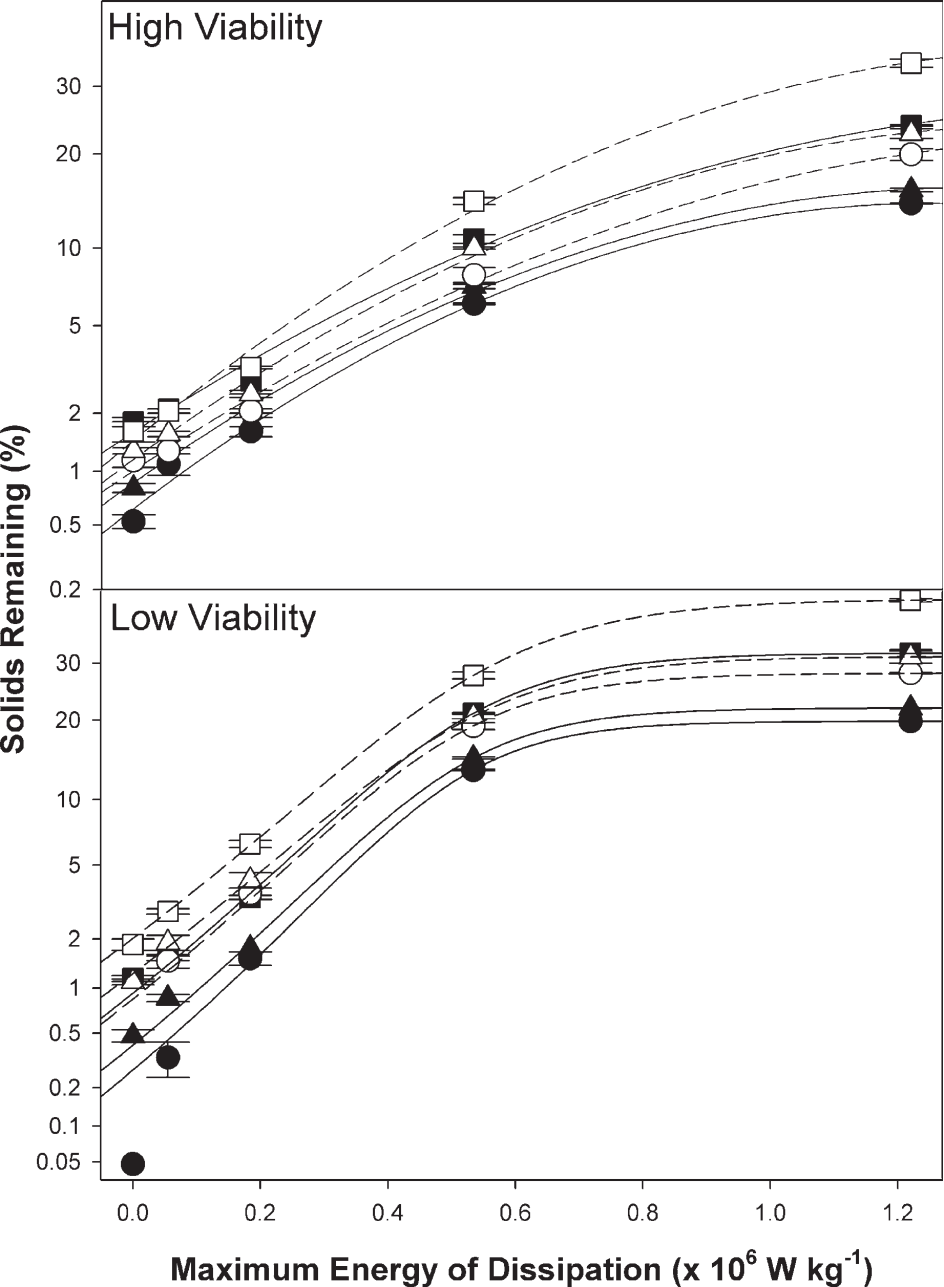
Effect of mild hypothermic culture conditions on centrifugation performance predicted using an USD model Samples taken from fed-batch cultures operated at 32 or 37°C were exposed to shear environments that represent those typically experienced in industrial scale centrifuges. The predicted clarification performance was determined by centrifugation of sheared and non-sheared samples in 96-well plates conditions to give V/c.t.Σ values of 1.88 (•,○), 2.56 (▲,Δ) and 5.00 × 10^−8^ m s^−1^ (▪,□), 32°C (filled symbols), 37°C (empty symbols).

Comparison of the centrifugation performance using a multiwell plate based method demonstrates how the difference in shear sensitivity between the two cultures observed in the cell viability data is translated into the solids remaining observed after centrifugation (Figure 6). Where the cell culture viability is high prior to centrifugation there is little difference observed in the solids remaining where no shear is applied, but on application of higher energy dissipation rates (equivalent to those observed in nonhermetically sealed centrifuge) a difference between the solids remaining in the supernatant from the different cultures and all V/c.t.Σ values measured was seen, rising to a difference of ∼4% under the highest shear conditions and worst clarification conditions. This poorer performance of the supernatant taken from the 37°C was also observed from the cultures taken at a low viability pre-shear, but is more pronounced.

**Figure 7:**
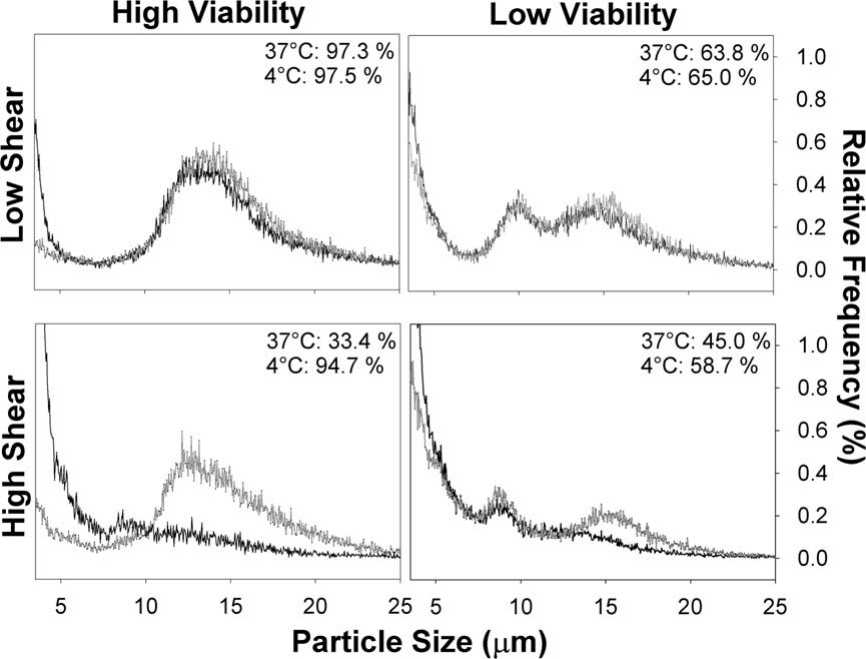
Impact of temperature reduction on particle size distribution following mimic of centrifugal shear forces Samples were taken on day 6 (high viability) or 12 (low viability) and were immediately processed (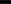) or adjusted to 4°C (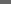) and held for 5 h prior to processing. Samples were sheared at 6,500 or 12,000 rpm, equivalent to maximum energy dissipation rates of 0.06 and 0.53 × 10^6^ W kg^−1^. The particle sizes were determined using the CASY analyzer, with the data shown in each plot the relative frequency of an average of five replicates. Viability, as determined using the Vi-Cell XR, is also indicated in each plot.

**Figure 8:**
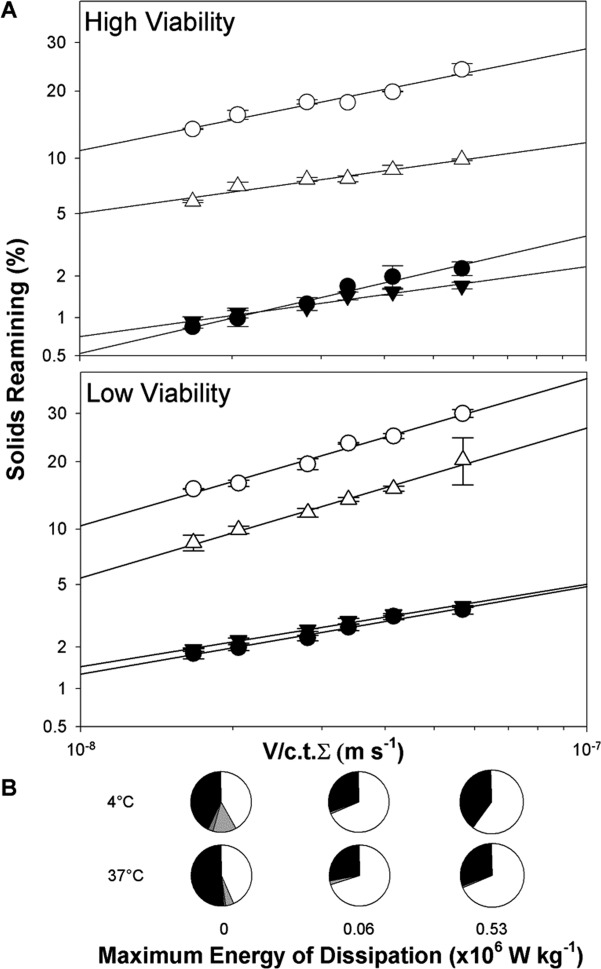
Impact of temperature reduction of cell culture material prior to centrifugation A: Samples were taken on days 6 (high viability) or 12 (low viability) and adjusted to the specified temperature. Once the target temperature was attained samples were sheared at 6,500 (○ for 37°C, Δ for 4°C) or 12,000 rpm (• for 37°C, ▲ for 4°C), equivalent to maximum energy dissipation rates of 0.06 and 0.53 × 10^6^ W kg^−1^. The samples were then centrifuged in conical bottom 96 deep round well plates at 3,000 rpm for 5 or 10 min, with different fill volumes to achieve V/c.t.Σ values of between 1.67 and 5.69 × 10^−8^ m s^−1^, equivalent to volumetric flow rates of 39 to 134 L h^−1^. B: Apoptosis was determined by flow cytometry. The viable (▪), early apoptotic (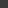), late apoptotic (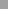), and nonviable cells populations (□) of the samples were determined on day 12.

To investigate further membrane fluidity properties on primary recovery performance, it was decided to perform centrifugation analysis of material at harvest temperature (i.e., 37°C) and reduced to 4°C for a shortened period considered insufficient for homeoviscous adaption. Although temperature reduction may not be appropriate for higher cell density cultures for which centrifugation performance would be adversely affected by viscosity effects, it was selected as both a means to demonstrate the impact of membrane rigidity because of temperature change and as a potential processing strategy, which would be readily implementable. As shown in Figures 7 and 8, temperature reduction has a dramatic effect on particle size post shearing and the resulting clarification levels achieved. Under high shear conditions, at both high and low viability, the viable cell population was significantly reduced when centrifugation was performed at standard harvest temperature, compared with reduced temperature. This is particularly evident at high viability, when cells are more susceptible to shear under standard conditions.^24^ Reducing the temperature of the cell culture material prior to centrifugation protects this population, increasing the viable cell population from 33.4 to 94.7% at high viability and under high shear conditions, and results in improved clarification levels. Apoptosis analysis of the sheared samples (data not shown) shows that the early apoptotic and late apoptotic populations, both of which are within the viable cell population are either completely lost or significantly reduced under any of the energy dissipation rates investigated and either temperature. This indicates that the apoptotic cells are therefore the most sensitive sub-set of cells to shear.

The adaption of cells to temperature reduction, either under long-term mild hypothermic conditions (i.e., homeoviscous adaption) as part of a cell culture strategy or as a short term means to improve primary recovery performance using a short-term pronounced temperature reduction has been shown to beneficial to centrifugation performance. The potential improvement associated with this change may enable centrifuge operation at a higher flow rate or a reduction in the capacity of subsequent steps such as depth filtration to achieve a similar level of clarification to the standard temperature culture conditions.

## Conclusions

Implementation of mild hypothermic conditions during culture prolonged the stationary phase, delayed the progression into apoptosis, and maintained higher viability. These effects were associated with increases in volumetric productivity. The glycosylation profile of the mAb used in this work was affected by culture time, but not significantly by the temperature conditions selected. Despite the increase in product titer on day of harvest when adopting a process using mild hypothermic conditions, the potential impact of implementing such changes further downstream is a significant consideration at process scale. Evaluation of HCP impurities by ELISA, SELDI-TOF MS and 2D-PAGE indicated that there were differences in impurity profiles, which may affect purification performance. Cellular adaption to growth at reduced temperature or short-term reduction immediately prior to primary recovery lowered the susceptibility of the cells to shear stress and we demonstrated that this affected process scale centrifugation, improving primary recovery performance.

In this work, by applying of a number of process and analytical characterization methods, we have demonstrated the close relationship between upstream and downstream operations and the value of improving their integration during process development. The knowledge gained from evaluations similar to those performed here may therefore be supplementary to the cost of goods analyses, which typically help to determine the value of novel cell culture strategies in biopharmaceutical manufacture and provide further support when deciding on their implementation.
